# Dermatophytes in Argentina: species distribution, comparative identification accuracy of conventional and proteomic methods, introduction of a novel molecular identification approach, and antifungal susceptibility profiles

**DOI:** 10.1128/aac.00796-26

**Published:** 2026-10-01

**Authors:** Natalia Sasoni, María Sol Barbagelata, Ivana Maldonado, Luciana Farias, Matias Cabeza, Manuela Loaiza-Oliva, María Gabriela Latorre Rapela, Soledad Gamarra, Norma Fernández, Guillermo García-Effron

**Affiliations:** 1 Cátedra de Parasitología y Micología - Facultad de Bioquímica y Ciencias Biológicas, Universidad Nacional del Litoral 28247, Santa Fe, Argentina; 2 Laboratorio de Micología y Diagnóstico Molecular - Instituto de Salud y Ambiente (ISAL), Consejo Nacional de Investigaciones Científicas y Técnicas (CONICET) 62873, Santa Fe, Argentina; 3 Sección Microbiología, Laboratorio Central, Hospital Alemán 62862, Buenos Aires, Argentina; 4 Laboratorio de Micología, División Infectología, Hospital de Clínicas José de San Martin, Catedra de Microbiología Clínica, Facultad de Farmacia y Bioquímica, Universidad de Buenos Aires 28196, Buenos Aires, Argentina; University Children's Hospital Münster, Münster, Germany

**Keywords:** MALDI-TOF, susceptibility testing, dermatophytes, Molecular identification

## Abstract

Dermatophytosis is a frequent superficial fungal infection, yet data from Argentina remain scarce. In this multicenter study, 127 isolates consecutively collected in 2024 were analyzed using morphology, matrix-assisted laser desorption/ionization time-of-flight mass spectrometry, internal transcribed spacer sequencing, and a novel multiplex PCR assay for rapid identification. Predominant species included *Trichophyton tonsurans*, *Trichophyton rubrum*, and the *Trichophyton mentagrophytes* complex. Antifungal testing (CLSI M38) revealed interspecies variability: terbinafine was most active, itraconazole showed better efficacy against *Microsporum canis*, while fluconazole displayed limited activity.

## INTRODUCTION

Dermatophytosis is the most common superficial fungal infection worldwide, caused by keratinophilic fungi that infect keratinized tissues such as skin, hair, and nails, producing clinical manifestations collectively referred to as tinea (1, 2). Clinical presentation varies by infecting species, host characteristics, and geographic region (1, 3). Despite its high global prevalence, data on dermatophyte species distribution and antifungal susceptibility in South America, particularly in Argentina, remain limited (4).

Accurate and timely identification of dermatophytes is essential for guiding appropriate antifungal therapy. Conventional identification methods that rely on macroscopic and microscopic morphology are inherently slow and may lack precision, particularly within closely related species such as the *Trichophyton mentagrophytes/Trichophyton interdigitale* complex (5). Advanced techniques, including matrix-assisted laser desorption/ionization time-of-flight mass spectrometry (MALDI-TOF MS) and ribosomal DNA (rDNA) sequencing, have improved both the speed and accuracy of dermatophyte identification (5, 6).

We analyzed 127 dermatophyte isolates consecutively collected between January and September 2024 from patients diagnosed with dermatophytosis in Argentina. Samples were obtained from two hospitals in Buenos Aires and two academic laboratories located in Buenos Aires and Santa Fe cities. Both male and female patients were included. Clinical diagnoses associated with the isolates were tinea unguium (26.3%), tinea pedis (23.7%), tinea capitis (21.2%), tinea corporis (16.1%), tinea cruris (1.7%), and 11.0% of cases with unknown clinical origin.

All isolates were cultured on Potato Dextrose Agar supplemented with chloramphenicol and on Lactrimel (Agar Borelli) medium at 28°C to evaluate macroscopic characteristics and pigment production. Physiological tests such as growth at 37°C, cycloheximide growth, urease, Bromocresol purple-milk solids glucose agar (BCP-MSG), hair perforation, and vitamin requirement were performed. Microscopic examination was performed using lactophenol cotton blue.

All isolates were identified using a comprehensive strategy that combined morphological, phenotypic, and physiological testing with MALDI-TOF MS with both Bruker Daltonics (Germany) and Shimadzu Axima systems (Japan), internal transcribed spacer (ITS) sequencing (4, 5), and a novel multiplex PCR assay targeting ITS polymorphisms. Importantly, the multiplex PCR assay was validated in a blinded fashion across the three participating laboratories to ensure reproducibility and reliability.

**Fig 1:**
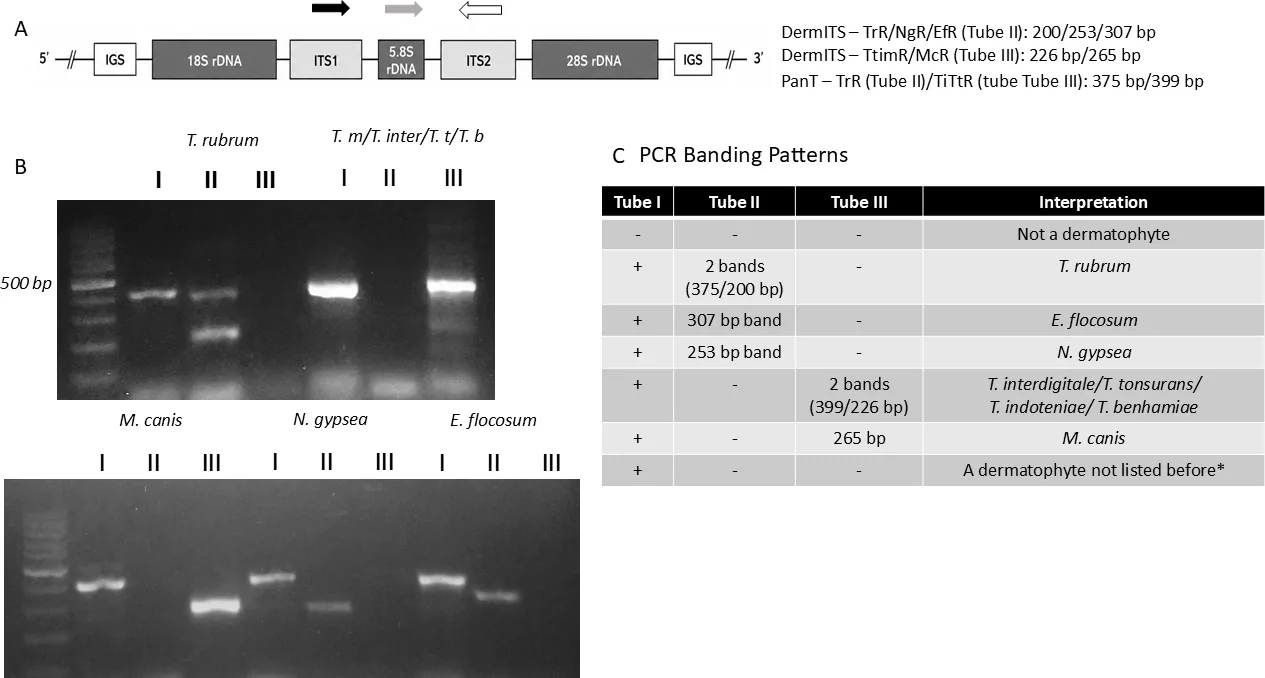
ITS-PCR amplification patterns for dermatophyte identification. (**A**) Schematic representation of the rDNA region showing IGS, 18S, ITS1, 5.8S, ITS2, 28S, and IGS. Roman numerals indicate reaction tubes. Tube I includes the primer set panDerm1/panDerm2, designed to amplify *chitin synthase I* of dermatophytes, serving as a universal control (7). Forward primers PanT (black arrow) and DermITS (gray arrow) target *Trichophyton* spp. and conserved dermatophyte regions within ITS1 and 5.8S, respectively, and were included in both tubes II and III. When combined with species-specific reverse primers (white arrow), these yielded amplicons of distinct sizes (with PanIT, 375–399 bp; with DermITS, 200–307 bp). (**B**) Agarose gel electrophoresis of ITS-PCR products from representative isolates. Left panel: *T. rubrum*, *T. mentagrophytes*, *T. interdigitale*, and *T. tonsurans*. Right panel: *M. canis*, *N. gypsea*, and *E. floccosum*. Molecular size marker (500 bp) is shown at the left of each gel. (**C**) Multiplex PCR banding patterns for the identification of dermatophytes. *Tested: *Trichophyton verrucosum* NR_154982.1, *Trichophyton concentricum* NR_144903, *Trichophyton soudanense* NR_155948.1, *Trichophyton schoenleinii* NR_153261, *Arthroderma* (*Trichophyton*) *terrestre* NR_189773.1, *Trichophyton erinacei* NR_149340.1, *Paraphyton* (*Microsporum*) *cookei* NR_155665.1, *Nannizzia lorica* (*Microsporum racemosum*) NR_178109.1, *Lobophyton* (*Microsporum*) *gallinae* NR_144882.1, and *Microsporum audouinii* NR_144883.1.

The multiplex PCR assay was developed (Fig. 1) as a proof of concept, designed to enable accurate species identification within 3 h and thereby eliminate the need for sequencing. The three-tube format allows rapid identification of major dermatophytes while grouping members of the *T. mentagrophytes* complex that share common ITS sequences (*T. mentagrophytes/interdigitale*, *Trichophyton tonsurans*, and *Trichophyton indotineae*). The tube sets used are as follows:

- Tube I: Primers amplifying dermatophyte *chitin synthase 1* (PanDerm1 and PanDerm2: 366 bp), designed to discriminate dermatophytes (positive PCR) from non-dermatophytes (negative PCR) (7).
- Tube II: Primers targeting ITS region combine two forward oligonucleotides—PanT (5′-CCCGCCGGAGGACAGAC-3′), which hybridizes to a conserved region of *Trichophyton* spp., and DermITS (5′-TCTTTGAACGCACATTGCGCC-3′), which binds a conserved region across all dermatophytes—with species-specific reverse primers for *Trichophyton rubrum* (TrR: 5′-TTCAGCGCCCTCAGGACCG-3′; 200/375 bp), *Epidermophyton floccosum* (EfR: 5′-GTCAACCTGAATGCGGAGGG-3′; 307 bp), and *Nannizzia gypsea* (NgR: 5′-TGATTGTAGGGAAGTTCATCCC-3′; 253 bp). Thus, *T. rubrum* yielded two bands (375 bp with PanT+TrR and 200 bp with DermITS+TrR), *E. floccosum* produced a single band (307 bp with DermITS+EfR), and *N. gypsea* generated one band (253 bp with DermITS+NgR).
  Tube III: This included PanT and DermITS, combined with reverse primers for *Microsporum canis* (McR: 5′-CAACCTGAATAATACATGGTGC-3′; 265 bp) and for *T. interdigitale*, *T. indotineae*, and *T. tonsurans* (TiTtR: 5′-AGATTTTGAGGCCAGGGCGG-3′; 226/399 bp). Accordingly, *T. interdigitale*, *T. tonsurans*, and *Trichophyton benhamiae* yielded two bands (399 bp with DermITS+TiTtR and 226 bp with PanT+TiTtR). *T. indotineae* would be expected to produce the same pattern based on its ITS sequences (GenBank accessions: *T. interdigitale* KJ722759.1, *T. tonsurans* AY213690.1, and *T. indotineae* PZ007874.1), although no strains were tested. In contrast, *M. canis* produced a single band (265 bp with DermITS+McR).

Thus, the rationale for employing three tubes is that interpretation depends not only on amplicon size but on the distinct banding patterns obtained across the three reactions, ensuring consistent and reliable species differentiation (Fig. 1).

Using combined classical, proteomic, and molecular approaches, the following species were identified: *Trichophyton tonsurans* (*n* = 38; 29.9%), *T. rubrum* (*n* = 35; 27.6%), *T. mentagrophytes* complex (*n* = 35; 27.6%), *Microsporum canis* (*n* = 7; 5.5%), *Nannizzia gypsea* (*n* = 6; 4.7%), *Epidermophyton floccosum* (*n* = 5; 3.9%), *Microsporum nannum*, and *T. benhamiae* (*n* = 1 each; 0.8%).

Morphological and physiological identification showed high concordance with proteomic and molecular methods for most species. Nevertheless, four isolates (3.1%) were misclassified by morphology alone. Two strains initially identified as *T. tonsurans* were reclassified as *T. rubrum* by sequencing and multiplex PCR, while two isolates assigned to the *T. mentagrophytes* complex were reclassified as *T. interdigitale* and *T. benhamiae* by MALDI-TOF MS. Overall, the multiplex PCR assay demonstrated 100% concordance with sequencing, whereas multiplex PCR methods showed 98.4% concordance with MALDI-TOF MS. The few discrepancies between proteomic and molecular approaches were confined to zoophilic members of the *T. mentagrophytes* complex (*T. interdigitale*) and *T. benhamiae*, which share identical ITS amplicon length and therefore cannot be reliably distinguished by the proposed multiplex PCR alone. Antifungal susceptibility testing was performed following CLSI M38 guidelines (8). The antifungal agents evaluated—fluconazole, clotrimazole, terbinafine, and itraconazole—were selected based on treatment commonly used in Argentina. Minimum inhibitory concentrations (MICs) were determined in triplicate, and geometric means were calculated.

Overall, terbinafine and itraconazole exhibited the strongest *in vitro* activity, with consistently low MIC values across all dermatophyte species (0.009 to 0.250 µg/mL and from 0.055 to 0.155 µg/mL, respectively). In contrast, fluconazole showed the highest MICs (16 to 128 µg/mL), whereas clotrimazole displayed intermediate values (0.096 to 1.000 µg/mL) (Table 1). The MIC profiles of terbinafine, itraconazole, and fluconazole closely mirror those reported previously in Argentina (Misiones), albeit in a smaller data set of strains, particularly for *T. tonsurans* (4). Interspecies variability in antifungal susceptibility was evident. Itraconazole consistently showed low MICs across all species, with only a modest one twofold increase in *N. gypsea*. Terbinafine was highly active against *Trichophyton* spp., whereas *M. canis* proved the least susceptible species to this allylamine, in line with previous reports (9, 10). Comparative fold analysis underscored this reduced susceptibility of *M. canis*, with values exceeding those of *N. gypsea* (4.05-fold), *T. rubrum* (8.33-fold), *T. mentagrophytes* complex (6.90-fold), and *T. tonsurans* (13.85-fold). In contrast, fluconazole exhibited the highest MICs (16–128 µg/mL), reinforcing the idea of reduced *in vitro* efficacy (9, 10). Notably, *Nannizzia gypsea* and *Trichophyton benhamiae* exhibited comparatively higher MIC values across multiple agents, suggesting decreased susceptibility, although only a limited number of isolates were analyzed. No terbinafine resistance was detected; however, continuous surveillance is mandatory, as cases of dermatophyte resistance have recently been reported in Uruguay (11). This is particularly relevant given the geographic proximity.

**TABLE 1:** Antifungal susceptibility profiles of dermatophyte species from Argentina^*a*^

Organism/drug	*n*	GM	MIC (µg/mL)								
FLC			>64	64	32	16	8	4	2	1	0.5
*Trichophyton rubrum*	34	34.7	15	**9**	3	2	2	3			
*Trichophyton tonsurans*	38	35.7	15	**7**	3	4	4	4	1		
*Trichophyton mentagrophytes* complex	35	72.1	**18**	11	4		1		1		
*Trichophyton benhamiae*	1	128.0	1								
*Epidermophyton floccosum*	5	64.0		5							
*Microsporum canis*	7	105.0	5	2							
*Microsporum nannum*	1	16.0				1					
*Nannizzia gypsea*	6	114	5	1							

ITC			0.5	0.25	0.12	0.06	0.03	0.015	0.007	0.003	0.0015
*Trichophyton rubrum*	34	0.057	1	2	7	**15**	4	4		1	
*Trichophyton tonsurans*	38	0.079		3	**17**	12	6				
*Trichophyton mentagrophytes* complex	35	0.055	1	4	8	**5**	11	6			
*Trichophyton benhamiae*	1	0.007							1		
*Epidermophyton floccosum*	5	0.080		1	1	2	1				
*Microsporum canis*	7	0.074		1	2	2	2				
*Microsporum nannum*	1	0.12			1						
*Nannizzia gypsea*	6	0.155		3	2	1					

CLZ			1	0.5	0.25	0.12	0.06	0.03	0.015	0.007	0.003
*Trichophyton rubrum*	34	0.158	2	6	**12**	6	4	1	1	2	
*Trichophyton tonsurans*	38	0.15	2	12	**8**	4	6	2	3	1	
*Trichophyton mentagrophytes* complex	35	0.096	2	5	5	**8**	3	6	6		
*Trichophyton benhamiae*	1	1	1								
*Epidermophyton floccosum*	5	0.435	1	2	2						
*Microsporum canis*	7	0.101	1		1	2	1	1	1		
*Microsporum nannum*	1	0.250			1						
*Nannizzia gypsea*	6	0.497	3	1	1	1					

TRB			0.5	0.25	0.12	0.06	0.03	0.015	0.007	0.003	0.0015
*Trichophyton rubrum*	34	0.015				3	14	**8**	4	1	4
*Trichophyton tonsurans*	38	0.09				2	4	**15**	9	4	4
*Trichophyton mentagrophytes* complex	35	0.018	2	1	1	3	8	**5**	11	4	
*Trichophyton benhamiae*	1	0.06				1					
*Epidermophyton floccosum*	5	0.038			1	1	2	1			
*Microsporum canis*	7	0.124	1		3	3					
*Microsporum nannum*	1	0.25		1							
*Nannizzia gypsea*	6	0.03				2	2	2			

^*a*^ *n*, number of strains of each dermatophyte species (identified by proteomic and molecular techniques). Under the name *T. mentagrophytes* complex, both *T. mentagrophytes* and *T. interdigitale* were included. CLZ: clotrimazole; FLC: fluconazole; TRB: terbinafine; ITC: itraconazole. Numbers in bold represent MIC₅₀, and underlined values indicate MIC₉₀ (data were obtained only for *T. rubrum*, *T. tonsurans*, and the *T. mentagrophytes* complex due to the limited number of isolates from other species). Off-scale MICs were adjusted to the next higher or lower concentration to calculate geometric means (GMs). Evaluated ranges: CLZ, TRB, and ITC: 1–0.0015 µg/mL; FLC: 64–0.125 µg/mL.

From a diagnostic perspective, morphological identification remains useful but may be insufficient for accurate differentiation within species complexes. The incorporation of MALDI-TOF MS and molecular methods significantly improves species-level identification (5, 6). The multiplex PCR assay developed in this study provides a major diagnostic advantage by enabling species-level identification without the need for extensive culture development, including conidiation, and even in pleomorphic cultures. Morphological identification requires fructification, and although MALDI-TOF MS can be performed prior to fructification, its accuracy may vary depending on the age and quality of the culture. In contrast, the PCR assay yields consistent results independent of culture duration, thereby overcoming these limitations. Identification using the multiplex PCR assay presented in this study was successfully achieved after only 4 days of growth, despite the absence of conidiation or any phenotypic traits, thereby demonstrating its robustness and reliability (data not shown). This approach markedly reduces diagnostic turnaround time and ensures reliable identification of dermatophytes.

Our study, based on a large multicenter data set (127 consecutive isolates) and a comprehensive identification strategy, demonstrates the diagnostic and clinical value of a novel multiplex PCR assay. The consistently low MICs for terbinafine and itraconazole across major dermatophyte species support their role as first-line systemic agents in Argentina, while the high MICs for fluconazole reinforce its limited utility—an observation that requires confirmation in clinical trials. The reduced susceptibility of *Microsporum canis* to terbinafine highlights the importance of accurate species-level identification, which the multiplex PCR assay achieves rapidly and reliably, even in pleomorphic cultures. Although limited by the absence of treatment history, a restricted antifungal panel, and reduced representation of rare species, the data set establishes the first large-scale baseline for antifungal susceptibility in Argentina. Importantly, the absence of terbinafine resistance in our cohort is reassuring, yet recent reports from neighboring Uruguay (11) underscore the need for ongoing surveillance.

In conclusion, these findings provide actionable guidance for clinicians, optimize antifungal selection, and introduce a diagnostic tool that shortens time to targeted therapy, thereby contributing to optimizing antifungal selection, improving patient outcomes, and establishing a framework for resistance surveillance in South America.
